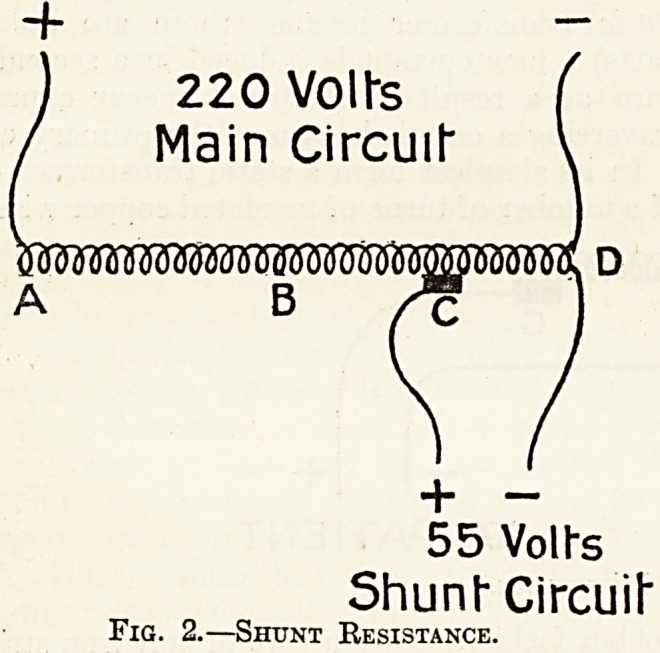# Current from the Main

**Published:** 1912-02-17

**Authors:** Alfred C. Norman

**Affiliations:** House Surgeon at Sunderland and Durham County Eye Infirmary.


### ELECTRICITY IN MODERN MEDICINE.*

### VII.—Current from the Main.

By ALFRED C. NORMAN, M.D. Edin., House Surgeon at Sunderland and Durham County Eye Infirmary.

### ALTERNATING CURRENT (*continued from p.* 430.)

FOR *x*-ray work it is necessary to send a current of from four to ten ampères in one direction only through the primary winding of the induction coil, and here again our best method of obtaining a continuous current is to use an alternating-current motor to drive a continuous-current dynamo. The "Pantostat" will not furnish sufficient ampèrage for the purpose, so a special motor-generator must be obtained for the *x*-ray room. There is, at any rate, one advantage in thus generating our own continuous current—we can choose our dynamo to give the voltage best suited to the purpose, and for ordinary *x*-ray work a pressure of 110 volts undoubtedly gives the most satisfactory results. The dynamo should be capable of furnishing at least 12 ampères of current at this pressure and to drive it we should require a motor of nearly 2 h.p. (the motor of course being wound specially to suit the periodicity and voltage of the alternating-current supply from the mains). Such a motor-generator, with motor and dynamo coupled together and mounted on one base, would cost about £35.

FIG. 1.—SERIES RESISTANCE.

The electro-magnets used in ophthalmic surgery can be excited only by a continuous current; hence, in hospitals with an alternating-current supply, when a large magnet is to be installed it is important to obtain a motor-generator (which may also be used for *x*-ray work) capable of giving sufficient current fully to excite the magnet. The 2-h.p. machine described above will be large enough for most of the magnets in use, but for Haab's giant magnet, which requires 16 ampères at 110 volts, it is necessary to obtain a 3-h.p. generator, and this would cost about £45. A machine of this size should be a fixture in, or near, the *x*-ray room and the current should be carried on stout cables to the theatre where magnet operations are performed.

Enough has been said to show that an alternating-current supply is not an unmixed blessing to the medical electrician, for suitable apparatus is both more complicated and more costly than in the case of the continuous-current supply.

#### STATIC TRANSFORMERS.

An exception to the above statement, however, is furnished by the alternating-current transformer, illustrated on page 429, which is eminently suitable for cautery work and for lighting small medical lamps. It is easily worked, its construction is simple, and it costs about £4. For general purposes and for moving about from ward to ward the writer strongly recommends a universal generator of the "Pantostat" type, but for an operating theatre or out-patient room where current for a cautery or a diagnostic lamp may be required at any moment this transformer makes a cheap and efficient fixture.

An alternating-current transformer depends upon the principle of induction. In the dynamo we had an example of induction in which current was generated as a result of mechanical motion; in the alternating-current transformer (generally known as a *static* transformer because there are no moving parts) a new current is induced in a second coil of wire as a result of electrical energy expended in traversing a circuit known as the primary coil.

In its simplest form a static transformer consists of a number of turns of insulated copper wire wound bobbin fashion round a core of soft iron strips, this being known as the primary coil. Round this primary coil, but having no electrical connection with it whatever, is wound more insulated wire, and this is known as the secondary coil. If a continuous current be passed through the primary coil nothing happens in the secondary while the current is flowing, but every time it is switched on or off or made to change its direction a momentary current will be induced in the secondary coil. Now an alternating current changes its direction many times in a second; therefore, if the primary coil be connected with an alternating-current supply a new alternating current will be induced in the secondary coil at every change of direction in the primary. If there are fewer turns of wire in the secondary than in the primary the voltage of the induced current will be lower than that of the supply, and the apparatus will be called a "step-down" transformer; whereas the voltage will be higher if there are more turns of wire in the secondary than in the primary, the apparatus then being known as a "step-up" transformer. An induction coil is a modified form of "step-up" transformer. For cautery work we always use a "step-down" transformer.

The efficiency of a good alternating-current transformer is often as high as 97 per cent., that is to say,

* Previous articles in this series have appeared in THE HOSPITAL of Nov. 11 and 25, Dec. 9 and 30, Jan. 13 and 27.

for every 100 watts passing through the primary we shall be able to obtain 97 watts at another voltage out of the secondary, for in proportion as the secondary current decreases in voltage it increases in ampèrage, and *vice versa* (an excellent example of the conservation of electrical energy). Now heat is produced entirely by ampèrage, and for cautery work we may require 18 ampères or more. If we were to obtain this amount of current directly from the 110-volt house mains (which it would be unsafe to do without special wiring and for several other reasons), we should consume 1,980 watts, but by using the alternating-current static transformer, shown on page 429, a current of 2 ampères at 110 volts (*i.e.* 220 watts) is transformed to 21 ampères at 10 volts (*i.e.* 210 watts). Thus we obtain a high ampèrage for our cautery, which is easily regulated on account of the low voltage, and since the transformer uses only two ampères from the main supply it is quite safe to connect it to any lamp-socket in the hospital. The transformer is contained in a

Fig. 2.—Shunt Resistance.

small cylinder mounted at the top of a slate slab, and below it are two variable resistances for controlling the current for cautery and lighting small lamps respectively. The one for light is made of fine wire, allowing the current to be cut down to less than 1 ampère. An alternating current produces a perfectly steady light in an incandescent lamp, provided the periodicity be not less than 40 per second. Static transformers are placed in every house supplied with the alternating current to reduce the very high voltage in the street mains to 200 or 100, but for medical purposes we have to reduce it still further. An interesting fact about static transformers is that practically no current flows through the primary when none is being taken from the secondary, owing to high inductive resistance.

### CONTINUOUS CURRENT.

In applying the alternating current we were confronted with two problems: the comparatively simple one of reducing the voltage, and the more difficult one of converting it into a continuous current for certain purposes. In the case of the continuous-current supply we are chiefly concerned in reducing the voltage (and in some cases increasing the ampèrage) so that the current may be safely used for medical purposes.

For galvanisation, electrolysis, and ionic medication, as well as for lighting small lamps, we may use some form of shunt rheostat or else a motor generator. For $x$-ray work, to control the current through the induction coil, a shunt resistance should be used for all voltages over 110; a series resistance for voltages up to 110. For cautery a motor-generator or an interrupter transformer must be used.

In a series resistance (or rheostat as it is sometimes called) all the current passing through the resistance coils also passes through the patient (or lamp or cautery as the case may be), that is to say, one of the wires leading to the patient is divided and the resistance interposed between its cut ends.

Fig. 1 shows the arrangement of a series resistance. One end of the cut wire from the battery is connected with the resistance wire at A, and the other with the sliding contact at C. While the sliding contact remains at C all the resistance wire is in the circuit and the current to the patient is limited, but when it is pushed along to B only half the resistance wire is traversed by the current (electricity always finds the shortest way home, therefore no currents traverse the coils of wire from B to C when it can get to the patient and back to the battery by leaving the resistance wire at B), hence the patient gets a stronger current. A series resistance should not be used with current from the main, except for $x$-ray work at 110 volts and less. In a shunt resistance a certain current flows through the main circuit and a variable portion of it is tapped off to form a shunt circuit.

Fig. 2 shows the usual connections of a shunt resistance. If A—D is a coil of wire having a total resistance of 44 ohms it will allow a current of 5 ampères to pass when it is connected directly to the 220-volt house mains. Current enters by the wire marked + and leaves by the wire marked −, thus forming the main circuit of the rheostat. But in passing from A to D (*i.e.* from the positive side to the negative) the voltage has dropped from 220 to 0, for obviously the negative pole can have no voltage; and if the resistance of the wire be uniform there will be a uniform drop all along the line. Now if a conducting wire (whose resistance may be ignored) be joined to a sliding contact at C, $\frac{3}{4}$ of the way along the resistance wire, and another conducting wire to D, and if these wires, forming a shunt circuit, be connected to a volt-meter we shall find that the voltage in the shunt circuit will be 55 (i.e., $\frac{1}{4}$ of the original 220 volts, for the current has already traversed $\frac{3}{4}$ of the resistance wire when it reaches C). If the sliding contact be pushed nearer to A the voltage in the shunt circuit will be increased; for instance, at B it will be 110 (i.e., $\frac{1}{2}$ of 220 since B is midway between A and D), and if it be moved towards D the voltage will be diminished in the shunt circuit until at D it will be 0. If, instead of a volt-meter, a patient be placed in the shunt circuit the amount of current he receives will depend upon the resistance of his skin and the voltage in the shunt circuit; the latter, as we have seen, can be varied by means of the sliding contact. Thus a shunt rheostat is really a volt selector.

*(To be continued.)*

## Figures and Tables

**Fig. 1. f1:**
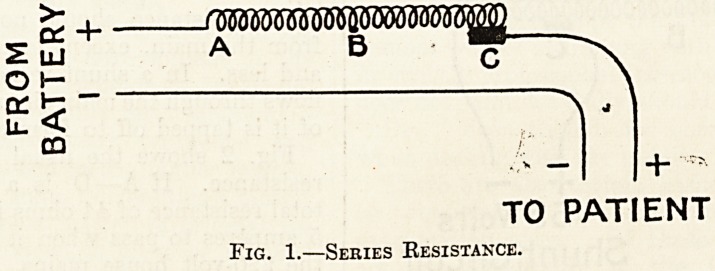


**Fig. 2. f2:**